# Pathways to loneliness: a mediation analysis investigating the social gradient of loneliness in persons with disabilities in Switzerland

**DOI:** 10.1186/s12939-021-01600-5

**Published:** 2021-12-20

**Authors:** Hannah Tough, Mirja Gross-Hemmi, Inge Eriks-Hoogland, Christine Fekete

**Affiliations:** 1 Swiss Paraplegic Research, Guido A. Zäch Strasse 4, 6207 Nottwil, Switzerland; 2 Department of Health Sciences and Medicine, University of Lucerne, Frohburgstrasse 2, 6207 Lucerne, Switzerland; 3 Swiss Paraplegic Centre, Guido A. Zäch Strasse 2, 6207 Nottwil, Switzerland

**Keywords:** Loneliness, Socioeconomic status, Spinal cord injury, Mediation, Psychosocial resources, Social isolation, Participation

## Abstract

**Background:**

The experience of loneliness can have drastic consequences for health and quality of life. Given that loneliness is highly prevalent in persons with physical disabilities and that loneliness more profoundly affects persons of low socioeconomic status, more evidence is required in order to understand the mechanisms determining loneliness in this population. The objective of this study is therefore to investigate the potential pathways through which socioeconomic status influences loneliness in persons with spinal cord injury.

**Methods:**

Mediation analysis utilising structural equation models and bias corrected and accelerated confidence intervals were used in order to test the mediation effects of health status, functioning, participation, social support and self-efficacy on the association between socioeconomic status and loneliness in persons with spinal cord injury. A latent construct was created for socioeconomic status with the indicators education, household income, financial hardship, subjective social status and engagement in paid work.

**Results:**

This study found evidence to support the mediating role of psychosocial resources and of secondary health conditions in the association between socioeconomic status and loneliness. The study demonstrated robust associations between socioeconomic status and all potential mediators, whereby higher socioeconomic status was associated with better health, participation and psychosocial resources, however, not all potential mediators were associated with loneliness. The serial mediation model explained the interplay between socioeconomic status, mediators on different levels, and loneliness. For example, emotional support and self-efficacy were both positively associated with fewer restrictions to participation (0.08 (CI: 0.05, 0.12); 0.29 (CI: 0.24, 0.36) respectively), and fewer restrictions to participation were found to be a result of improved functional independence and fewer secondary health conditions (0.23 (CI: 0.15, 0.39); − 0.29 (CI: − 0.36, − 0.20) respectively).

**Conclusions:**

Our findings highlight the vulnerability of persons with low socioeconomic status to loneliness in persons with spinal cord injury and identified potential mediating factors, such as health, functioning, participation and psychosocial resources, in the association between socioeconomic status and loneliness. This population-based evidence suggests potential targets of interventions on the pathway to loneliness, through which socioeconomic status influences loneliness. The complexity of the model shows the need for comprehensive interprofessional rehabilitation to identify and support people with lower socioeconomic status and concomitant risk factors for loneliness.

## Introduction

The higher prevalence of loneliness in groups with lower socioeconomic status (SES) may contribute to the emergence of health inequalities [1–5]. Not only is loneliness a stressful and negative experience in and of itself, but loneliness has also been identified as a risk factor for poor health behaviours [6], adverse health outcomes [7–9] and mortality [10, 11]. Given its relevance for health and its potential role in driving health inequalities, preventing loneliness in low SES groups is of high importance. Understanding the drivers of loneliness in persons with low SES may help to successfully target interventions. As SES is generally seen as an unmodifiable factor in public health interventions, more knowledge on modifiable factors on the pathway to loneliness is needed in order to address loneliness and therefore reduce resulting negative consequences on health. Therefore, a thorough understanding of mechanisms leading to loneliness is needed for targeted intervention planning, and insights into reasons for the higher prevalence of loneliness in groups with low SES is essential. Although those reasons are currently unclear, there are several theoretical pathways linking SES and loneliness.

**Fig. 1:**
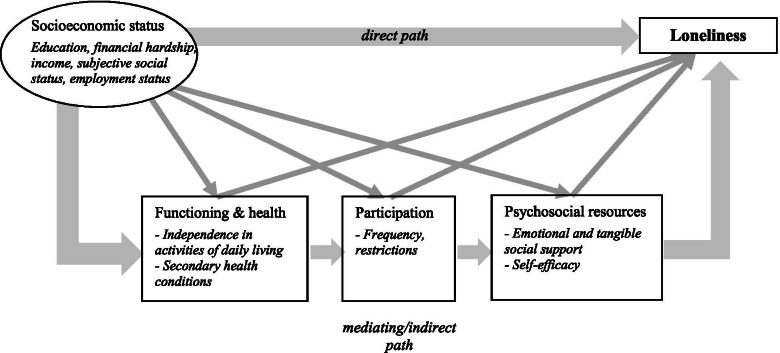
Serial mediation model linking socioeconomic status and loneliness

Low SES may create the conditions needed for loneliness to thrive. Low SES may act independently on different factors that potentially affect loneliness, such as the health status, functioning, participation and psychosocial resources. For example, low participation can be a direct outcome of low SES in case of lacking financial resources to engage in leisure time activities, or low self-esteem might be a direct consequence of poor SES and related feelings of marginalization. In contrast to this rather simplistic approach, a further approach maintains that poor SES leads to a *sequence* of poor outcomes that eventually increases the risk of loneliness (Fig. 1) [12]. This approach, also known as serial mediation model, assumes that SES is followed by a causal chain linking the mediators, with a distinct assumed direction of causal flow that affect loneliness [13]. For example, persons from low SES groups are at enhanced risk of poor health status or reduced functioning, which could lead to decreased participation, and reduced participation may have significant negative effects on psychosocial resources such as self-esteem and self-efficacy, ultimately increasing the risk of loneliness. Given the theoretical model which assumes a certain temporal ordering of the mediators used in this study [12], we assume causality to be predominantly unidirectional.

Our study including persons with a spinal cord injury (SCI) focuses on a population which is highly vulnerable to loneliness, as studies have shown that the prevalence of loneliness is often elevated in persons with a physical disability [14, 15]. Persons coping with physical disability may be exposed to additional risk factors for loneliness, such as restrictions in social participation due to negative societal attitudes, functional limitations and diverse environmental barriers [16]. Persons with physical disabilities may also become emotionally isolated from their existing social circle, especially if they feel that they are no longer understood or if they are living in an intimate relationship whereby one half is providing informal care [17, 18]. Our study focuses on persons with SCI in Switzerland. SCI offers an informative case in point, as it often leads to physical disability characterised by varying degrees of functional limitations, depending on the level and completeness of the spinal cord lesion. In Switzerland, the social participation of persons with SCI has been studied in different settings. Evidence suggest that individuals with SCI have decreased labour market participation [19], lower relationship satisfaction, live more frequently alone, and are more frequently single than the general population [20]. The main barriers reported by persons with a SCI are inaccessibility of buildings and public spaces and difficulties with transportation, barriers were more likely to be reported in persons with lower household income [21]. In a recent study of persons affected by SCI in Switzerland, subjective social status and experiences of financial hardship were found to have the highest discriminative power in terms of determining loneliness [22]. This supports the notion that loneliness is elevated in persons with SCI with low SES in Switzerland, and possible causes of this may be a higher perception of environmental barriers, fewer opportunities for social relationships, and lower social participation. The causal pathways leading to loneliness have however yet to be studied in more detail.

Given that loneliness is highly prevalent in persons with physical disabilities and that evidence on pathways linking SES and loneliness remains largely unexplored in this vulnerable population, the objective of this study is to investigate the potential pathways through which SES influences loneliness in persons with physical disabilities, namely SCI. A serial mediation model is developed in order to contribute to the understanding of underlying mechanisms linking SES and loneliness.

## Methods

### Participants

The study utilized cross-sectional data from the Swiss Spinal Cord Injury Cohort Study (SwiSCI), the second population-based SwiSCI community survey [23]. SwiSCI included community-dwelling persons aged over 16 years with traumatic or non-traumatic SCI (e.g. due to degeneration of the spinal column, tumor, vascular problem, or infection) living in Switzerland. We excluded people with congenital conditions leading to SCI, those with neurodegenerative disorders and Guillain-Barré syndrome. Participants were recruited based on records of Swiss Paraplegic Association members (organization representing people with SCI), ParaHelp (specified SCI home care organization) and all four specialized SCI-rehabilitation centers in Switzerland. This resulted in a source population of 4493 individuals (thereof 3959 eligible) who were invited to the survey. This study uses data from 1294 individuals, giving a response rate of 32.7%.

### Study design

The second SwiSCI community survey included two questionnaires that were sent to participants with an interval of 4-6 weeks. Data collection was performed between 3/2017 and 3/2018. A mixed-mode data collection design including paper-and-pencil or online questionnaire and face-to-face or telephone interviews was used to achieve optimal response rates. The questionnaires were provided in three official Swiss languages (German, French or Italian) and the English reference questionnaire is available online (https://swisci.ch/en/research-projects-home/study-design/community-survey). Further details on recruitment outcomes, participation rates, and non-response bias in the SwiSCI community survey 2017 can be found elsewhere [23].

### Measures

*Loneliness* was assessed using three items from the UCLA Three-Item Loneliness Scale (UCLA-SF) which captures subjective feelings of loneliness [24]. Participants were asked to indicate whether they feel that they lacked companionship, feel left out, and feel excluded in everyday life. Response options were on a five-point Likert scale from 0 ‘not at all’ to 4 ‘completely’ and a sum score ranging from 0 to 12 was built, with higher scores representing higher loneliness. The response options were adapted from the original scale, whereby there were three response options. This was as loneliness was assessed as part of a larger battery on psychosocial resources and it was decided to avoid introducing new response scales for different items to reduce participant burden whilst filling out the questionnaire. This scale has recently been validated in an SCI population and showed adequate metric properties [25]. Cronbach’s alpha was 0.75, demonstrating satisfactory internal consistency in our sample.

*Socioeconomic status*: Education, household income, financial hardship, subjective social status and employment status were used to operationalize SES. Education was assessed according to the International Standard Classification of Education as total years of formal education, combining school and vocational training [26] excluding potential re-training after SCI. Income was measured by net equivalent household income, including information on disposable income weighted by the number of adults and children in the household according to OECD criteria [27]. Financial hardship was measured with a single item asking participants about problems faced due to their financial situation, offering four response options (not applicable, had no influence, made my life a bit more difficult, made my life a lot more difficult) [28]. The MacArthur Scale of Subjective Social Status was used to capture the subjective evaluation of one’s position in society, represented by a 10-rung ladder [29]. A single dichotomous variable was used to assess involvement in paid employment (yes/no).

*Functioning and health: Functional independence* was measured using the self-reported version of the Spinal Cord Independence Measure (SCIM-SR) [30]. This instrument measures independence in performing activities of daily living, such as dressing and feeding oneself, performing transfers out of a wheelchair, and mobility within and outside the house. The sum score comprises the three subscales of self-care, respiratory and bowel management, and mobility. Each item was rated on a scale ranging from ‘I need total assistance’ to ‘I am completely independent’. Rasch transformed scores were used [31]. The scores range from 0 to 100, with higher scores representing higher functional independence. *Secondary health conditions* were measured using the Spinal Cord Injury Secondary Conditions Scale (SCI-SCS) [32]. A list of 14 secondary health conditions that are commonly diagnosed in people living with SCI were assessed with information on the presence and impact of health conditions. Self-report of impact is over the past three months and on a 4-point ordinal scale (0 ‘not existing or insignificant’; 1 ‘mild or infrequent’, 2 ‘moderate or occasional’, 3 ‘severe or chronic’). The health conditions which were assessed were: chronic pain, spasticity, circulatory problems, bladder dysfunction, bowel dysfunction, contractures, urinary tract infections, autonomic dysreflexia, postural hypotension, injury caused by loss of sensation, respiratory problems, pressure injuries, heterotopic ossification, and sleep problems. A sum score ranging from 0 to 42 was built for analysis, with higher scores indicating more secondary conditions.

*Participation* was measured with two subscales of the Utrecht Scale of Evaluation of Rehabilitation-Participation (USER-Participation), namely the frequency and restrictions scales [33]. The frequency scale (11 items) assesses the hours or occasions spent on productive, leisure and social activities and ranges from none at all/never to 36 h or more/19 times or more. The restrictions scale (11 items) assesses experienced restrictions on vocational, mobility, leisure and social activities due to one’s health condition and item scores range from 0 (not possible at all) to 3 (no difficulty at all). To assure linear metric properties for use in analysis, Rasch transformed scores were used for the restriction scale. The scores range from 0 to 100 with higher scores representing better participation (higher frequency, less restrictions). A Rasch analysis of the frequency scale (0-100) is not warranted [34], as different productive activities, such as pursuing paid work, doing housework, and volunteering work cannot be performed simultaneously, this renders the scaling of associated frequencies into a single dimension conceptually meaningless.

*Psychosocial resources* included emotional and tangible social support, and self-efficacy. General self-efficacy, which describes the general confidence in one’s own abilities to overcome difficulties, was assessed using a modified version of the General Self-Efficacy Scale (GSES) consisting of five items. Participants were asked to rate different statements, as for example ‘I can find a solution for every problem’ or ‘I know how to act in an unexpected situation’, on a four-point Likert scale ranging from 1 ‘not true’ to 4 ‘exactly true’. A sum score ranging from 5 to 20 was built, with higher scores indicating higher self-efficacy [35]. Social support was measured with three items on instrumental and three items on emotional support taken from the Swiss Health Survey [36]. Participants were asked to rate the extent of emotional and tangible support they receive from their partner, family, and friends if needed, on a numeric scale ranging from 0 ‘not at all’ to 10 ‘very much’. The scale included the option to indicate if a source of support was unavailable (e.g. not having a partner). A mean score ranging from 0 to 10 was calculated from scores of social support sources available, with higher mean score indicating higher level of social support.

### Statistical analysis

First, we describe basic sample characteristics and main variables of interest. Second, we employed a serial mediation model by utilizing structural equation modelling (SEM) in order to understand the pathways connecting SES with loneliness, while calculating the indirect and direct effects of SES on loneliness with the mediators of functioning and health, participation and psychosocial resources.

As a preparatory step for SEM, we used confirmatory factor analysis to validate the latent SES construct. In a next step, we investigated unadjusted regression coefficients between each of the potential mediators, the latent SES variable and the outcome variable loneliness. If both coefficients (between mediator and SES as well as between SES and loneliness) had a *p value* < 0.05, they were included in subsequent models as potential mediators, if not they were dropped from analysis. We also investigated the unadjusted indirect effects over each of the mediators independently and provide standardized coefficients for indirect effects along with bias-corrected CIs.

As a further preliminary analysis for the serial mediation model, we used a so-called parallel mediation model to derive adjusted regression coefficients of the direct paths between each of the potential mediators, the latent SES variable and the outcome variable loneliness. In this model, all covariances between the mediators were included. Only variables with relevant paths (*p value < 0.05)* were included in the main analysis of the serial mediation model. This resulted in the exclusion of the variables ‘participation frequency’ and ‘tangible support’ for the serial mediation model. The main SEM analysis assessed the potential ordering of mediators based on the conceptual model of serial mediation (Fig. 1). Again, we included covariances between the mediators to account for their highly correlated nature. Indirect effects estimate the effects of the antecedent variable SES on the outcome variable loneliness via the multiple mediators. Direct effects estimate the effect between SES and loneliness, when controlling for the mediators, and the total effect is the sum of both, the indirect and direct effect. Bias-corrected and accelerated bootstrapping with 5000 replications with replacements was used to enable the estimation of asymmetrical CIs for the indirect effects in mediation analysis and for multiple mediation models, whereby all mediators were included in one model [37]. Adequate model fit was assessed by a non-significant χ^2^ test (vulnerable to sample size), a comparative fit index (CFI) > 0.95, and the root mean square error of approximation (RMSEA) < 0.06. We report standardized regression coefficients and 95% CIs. SEM analysis is conducted on non-imputed data using full information maximum likelihood (FIML) estimation, which adequately accounts for missing data. Proportion of mediated effects was calculated. All analyses were conducted using STATA Version 16.0 for Windows (College Station, TX, USA) and R (R Core Team (2020)).

## Results

Table 1 profiles the study sample. The majority of participants were male (71%), mean age was 56.3 years, and on average participants lived for 18.8 years with SCI. Over one third were in paid work, and around one-quarter reported experiencing financial hardship, there was a mean of 14.3 years in formal education and a net household monthly income of 4446 CHF. The average score for functional independence was 74.6 on a 0-100 scale and participants reported a mean score of 14.1 for secondary health conditions on a 0-42 scale. On a scale from 0 to 12, participants had a mean score of 2.6 for loneliness, with similar levels of emotional and tangible support, with means of 7.0 and 7.2 respectively on a 0-12 scale. Mean frequency in participation was measured at 29.5, whereas restrictions was measured at 69.4 on 0-100 scales.

**Table 1:** Characteristics of the SwiSCI community survey 2017 population

Variables (% missing)		Total (*n* = 1283)	
*Demographic characteristics*		n (%)	*mean (SD); median (IQR)*
Gender (0)			
Male		910 (70.9)	
Female		373 (29.1)	
Age at time of survey in years (0)			56.3 (14.4); 57.0 (21.0)
16-30 yrs		54 (4.2)	
31-45 yrs		252 (19.6)	
46-60 yrs		440 (34.3)	
61-75 yrs		428 (33.4)	
76+ yrs		109 (8.5)	
Education (4.3)			
Compulsory schooling (≤ 9 yrs)		78 (6.4)	
Vocational training (10-12 yrs)		239 (19.5)	
Secondary education (13-16 yrs)		607 (49.4)	
University education (≥17 yrs)		304 (24.8)	
Employment (0)			
Not in paid work		791 (61.7)	
In paid work		492 (38.3)	
Financial hardship (2.9)			
No		955 (76.6)	
Yes		291 (23.4)	
Net household income (23.4)			4446.6 (1220.7); 3400.0 (1098.2)
Subjective social status (4.7)			5.6 (1.9); 6.0 (3.0)
*SCI characteristics*			
Years since injury (5.9)			
≤ 5 yrs		166 (13.8)	
6-15 yrs		440 (36.5)	
16-25 yrs		263 (21.8)	
26+ yrs		338 (28.0)	
Type of SCI (10.8)			
Paraplegia/Incomplete		481 (37.5)	
Paraplegia/Complete		326 (25.4)	
Tetraplegia/Incomplete		249 (19.4)	
Tetraplegia/Complete		88 (6.9)	
Cause of SCI (1.9)			
Traumatic		888 (70.5)	
Non-traumatic		371 (29.5)	
*Functioning & health*	*Range*		
Functional independence (SCIM-SR score) (12.3)	0-100		74.6 (11.6); 74.2 (10.7)
Secondary health conditions (SCS-SCI) (25.6)	0-42		14.1 (7.5); 14.0 (10.0)
*Participation (USER-P)*			
Restrictions (5.5)	0-100		69.4 (17.9); 68.0 (23.0)
Frequency (4.5)	0-100		29.5 (14.1); 30.0 (19.3)
*Psychosocial resources*			
Emotional support (1.9)	0-10		7.2 (2.3); 7.7 (3.7)
Tangible support (1.7)	0-10		7.0 (2.3); 7.0 (3.7)
Self-efficacy (GSES) (1.8)	1-4		3.1 (0.6); 3.0 (0.5)
*Loneliness*			
Loneliness (UCLA-SF) (1.6)	0-12		2.6 (2.6); 2.0 (4.0)

*Abbreviations*: *GSES* General Self-Efficacy Scale, *IQR* Interquartile range, *SCI* Spinal cord injury, *SCIM-SR* Spinal Cord Independence Measure for self-report, *SCS-SCI* Secondary Conditions Scale for Spinal Cord Injury, *SD* Standard deviation, *UCLA-SF* UCLA-short form, *USER-P* Utrecht Scale of Evaluation of Rehabilitation-Participation

Table 2 displays unadjusted and adjusted associations between SES and mediators, as well as between mediators and the outcome loneliness. It also includes results of the parallel mediation model and the indirect effects of each included mediator. In unadjusted analysis, all associations were relevant (*p* values < 0.05), except for participation frequency and loneliness. The indirect effects were largest for the mediators of self-efficacy and secondary conditions, which was reflected in the larger proportions of mediated effects. More specifically, nearly 34% of the effect between SES and loneliness was mediated by self-efficacy, while only around 7% of the effect between SES and loneliness was mediated by poor frequency of participation. Adjusted analysis from the parallel mediation model suggest that only three variables were responsible for mediation, namely that poorer emotional support, poorer self-efficacy and higher prevalence of secondary health conditions in persons with lower SES mediate the association of SES and loneliness (*p value < 0.05*). We found that the indirect effect from SES to loneliness via mediating factors and direct effects from SES to loneliness are comparable in size.

**Table 2:** Standardized unadjusted and adjusted coefficients (from the parallel mediation model) of associations between socioeconomic status (SES) and mediators, and between mediators and loneliness, including indirect effects of the different SES – mediator – loneliness paths

SES - > Mediator	Unadjusted Coefficient (95% CI)	Adjusted Coefficient (95% CI)	Mediator - > Loneliness	Unadjusted Coefficient (95% CI)	Adjusted Coefficient (95% CI)	Unadjusted Indirect Effect Std Estimate (95% Bootstrap CI)	Adjusted Indirect Effect Std Estimate (95% Bootstrap CI)
**Mediators - Psychosocial resources**							
SES - > emotional support	**0.46 (0.31, 0.61)**	**0.71 (0.49, 1.00)**	emotional support - > loneliness	**−0.33 (− 0.40, − 0.27)**	**− 0.25 (− 0.34, − 0.14)**	**− 0.15 (− 0.21, − 0.10)**	**−0.18 (− 0.29, − 0.09)**
SES - > tangible support	**0.41 (0.28, 0.55)**	**0.56 (0.35, 0.82)**	tangible support - > loneliness	**− 0.26 (− 0.31, − 0.20)**	−0.01 (− 0.10, 0.09)	**− 0.10 (− 0.15, − 0.07)**	− 0.01 (− 0.07, 0.07)
SES - > self-efficacy	**0.63 (0.49, 0.80)**	**1.04 (0.75, 1.45)**	self-efficacy - > loneliness	**− 0.39 (− 0.47, − 0.32)**	**−0.27 (− 0.34, − 0.19)**	**−0.24 (− 0.33, − 0.17)**	**−0.28 (− 0.43, − 0.19)**
**Mediators - Participation**							
SES - > restrictions	**0.71 (0.57, 0.89)**	**1.56 (1.13, 2.21)**	restrictions - > loneliness	**− 0.20 (− 0.26, − 0.15)**	−0.06 (− 0.14, 0.02)	**−0.14 (− 0.20, − 0.10)**	−0.10 (− 0.22, 0.04)
SES - > frequency	**0.75 (0.60, 0.94)**	**1.50 (1.08, 1.97)**	frequency - > loneliness	−0.06 (− 0.13, 0.00)	0.04 (− 0.02, 0.12)	− 0.04 (− 0.10, 0.00)	0.06 (− 0.04, 0.18)
**Mediators – Functioning and health**							
SES - > secondary conditions	**−0.82 (−1.03, − 0.62)**	**−1.39 (− 1.96, − 0.99)**	secondary conditions - > loneliness	**0.26 (0.20, 0.33)**	**0.10 (0.02, 0.17)**	**−0.21 (− 0.30, − 0.14)**	**−0.13 (− 0.24, − 0.02)**
SES - > functional independence	**0.53 (0.39, 0.70)**	**1.20 (0.84, 1.70)**	functional independence - > loneliness	**−0.16 (− 0.22, − 0.10)**	0.02 (− 0.06, 0.08)	**−0.09 (− 0.13, − 0.05)**	0.02 (− 0.07, 0.12)

*Abbreviations*: *C* confidence interval. **Bold coefficients** indicate significant associations with CIs not crossing 0 (*p* value < 0.05)

Given that the indirect effect of participation frequency was not significant in unadjusted as well as adjusted analysis (Table 2), this potential moderator was excluded from the serial mediation model. Further, as the serial mediation model assumes psychosocial resources to form the last level of mediation, tangible support is also excluded from serial mediation as the assumed path between tangible support and loneliness was also not significant.

**Fig. 2:**
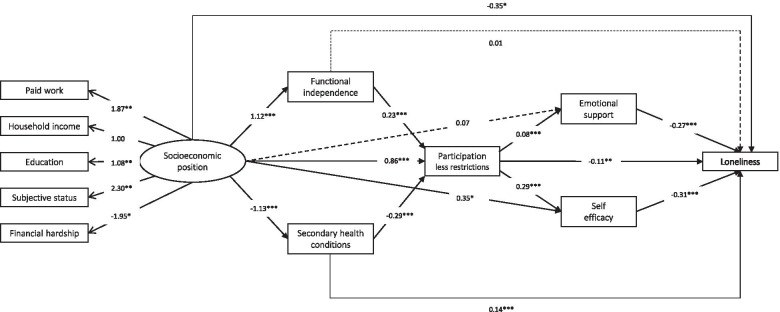
Serial mediation model on the association between socioeconomic status and loneliness including the mediators functioning and health, participation and psychosocial resources. Numbers indicate path coefficients. Dashed lines indicate non-significant paths, continuous lines indicate significant paths (*p < 0.05; **p < 0.01; ***p < 0.001)

In the serial mediation model, the large majority of hypothesized paths between different variables were relevant (p value < 0.05; Fig. 2). The largest indirect effect over mediators on all levels was seen in the path of SES over secondary conditions, restrictions in participation and self-efficacy to loneliness (Std estimate − 0.03, 95% bootstrap CI -- 0.05, − 0.02) (Table 3). The largest indirect effects over single mediators were observed for secondary conditions (Std estimate − 0.16, 95% bootstrap CI -- 0.33, − 0.07) and self-efficacy (Std estimate − 0.11, 95% bootstrap CI -- 0.25, − 0.02). The model had a reasonable fit.

**Table 3:** Standardized indirect, direct, and total effects of socioeconomic status on loneliness in the serial mediation model

Indirect effects	Std estimate	95% bootstrap CI (Bold CI indicate those not crossing 0)
**Mediators on all levels**		
SES- > functional independence - > participation restrictions - > self-efficacy - > loneliness	**− 0.023**	**− 0.074, − 0.009**
SES- > functional independence - > participation restrictions - > emotional support - > loneliness	**− 0.006**	**− 0.021, − 0.002**
SES- > secondary conditions - > participation restrictions - > self-efficacy- > loneliness	**− 0.029**	**− 0.050, − 0.015**
SES- > secondary conditions - > participation restrictions - > emotional support - > loneliness	**− 0.007**	**− 0.014, − 0.003**
**Mediators on two levels**		
SES- > functional independence - > participation restrictions - > loneliness	**− 0.029**	**− 0.101, − 0.007**
SES- > participation restrictions - > emotional support - > loneliness	**− 0.019**	**− 0.038, − 0.009**
SES- > secondary conditions - > participation restrictions - > loneliness	**− 0.037**	**− 0.069, − 0.012**
SES- > participation restrictions - > self-efficacy - > loneliness	**− 0.077**	**− 0.134, − 0.043**
**One mediator**		
SES- > functional independence - > loneliness	0.016	− 0.066, 0.104
SES- > secondary conditions - > loneliness	**− 0.160**	**− 0.330, − 0.072**
SES- > participation restrictions - > loneliness	**− 0.096**	**− 0.188, − 0.030**
SES- > self-efficacy - > loneliness	**− 0.108**	**− 0.250, − 0.021**
SES - > emotional support - > loneliness	− 0.020	− 0.074, 0.025
**Total indirect effect**	**− 0.591**	**− 1.077, − 0.369**
**Total direct effect**	**− 0.348**	**−0.781, − 0.016**
**Total effect**	**− 0.939**	**− 1.697, − 0.521**
**Proportion mediated effect**	62.9%	
**Model fit**		
X2	453.8 *(p value < 0.001)*	
CFI	0.91	
RMSEA	0.083	0.00, 0.085

*Abbreviations*: *CI* Confidence Interval, *CFI* Comparative Fit Index, *RMSEA* Root Mean Square Error of Approximation

## Discussion

This study aimed to understand the pathways through which SES influences loneliness in a large sample of persons with physical disabilities, namely SCI. In order to do this, a serial mediation model was developed with a number of potential mediator variables selected from the literature and from previous analyses. This study highlighted the importance of secondary conditions, functional independence, participation restrictions and the psychosocial resources emotional support and self-efficacy in the association with loneliness. More specifically, we observed that both, improved functional independence and fewer secondary conditions, were related to fewer participation restrictions and that fewer participation restrictions were subsequently related to increased emotional support and self-efficacy, which were finally linked to decreased loneliness. Our findings also highlighted the robust associations between SES and all potential mediators in our study, demonstrating the influence of SES on diverse areas of life, from health to psychosocial resources, which ultimately shape the extent of experienced loneliness.

This study demonstrated robust associations between SES and all potential mediators, in both unadjusted as well as the adjusted parallel and serial mediation analysis (except for the association SES and emotional support). This study therefore provides evidence to support the notion that there is a social gradient to many aspects of everyday life, from health status, to participation [38, 39], to the availability of psychosocial resources [40] that ultimately contribute to the social gradient in loneliness. Although we see that SES impacts on all of the potential mediators, only secondary health conditions, emotional support and self-efficacy were found to have robust mediation effects in adjusted analysis of the parallel mediation model. The mediating role of secondary health conditions and psychosocial resources in the SES-loneliness association has previously been found in the caregivers of persons with SCI [41] and more generally in persons with physical disabilities [42]. This highlights the importance of psychosocial resources, but also hints to the fact that there is a potential interplay between mediating factors on the pathway to loneliness, as mediation results changed after adjustment. This therefore reinforces the need to explore this interplay more thoroughly in the serial mediation model. Until now, a serial approach trying to link SES and loneliness via a sequence of mediators has not been explored in the context of SCI, and our study suggests that persons with physical disabilities who are socially deprived in terms of low SES, suffer from a double burden or an accumulation of risk factors for loneliness. For example, the functional dependence and participation restrictions due to the physical disability may create additional risk factors for loneliness besides the well-known loneliness risk factors associated with low SES. Moreover, the importance of psychosocial resources and the qualitative aspects of social relationships for loneliness, and more broadly for wellbeing, has previously been reported for general populations and populations with SCI [12, 14, 43, 44]. Our study contributes to this evidence by suggesting that quantitative elements of social relationships, such as frequency of participation play a minor role in the creation of loneliness, but that the qualitative aspects of social relationships, such as perceived participation restrictions and the perception of emotional support were important on the pathway to loneliness. Although social support and self-efficacy have long been recognized as resources for the adaptation process after the SCI onset and the maintenance of health in the long-term [45–47], studies disentangling the complex interplay between SES, psychosocial resources and loneliness are currently missing.

The fact that we found significant indirect effects involving mediators or several levels, and also that associations between mediators were prominent provides evidence for the “filtration model” as proposed by Hawkley et al. [12, 48]. This model suggests that “distal” elements, such as SES and sociodemographic characteristics, shape an individual’s social structures, such as their participation in social networks that ultimately influences more “proximal” factors, such as the quality of their social relationships and their psychosocial resources. The conceptual model devised by Hawkley et al. was strengthened by our findings in the serial mediation model as the majority of the hypothesized paths between SES, mediators and loneliness were significant. Providing evidence that upstream factors, shaped by the differing opportunities presented to individuals of differing social standing, influence an individual’s participation in their social environment [39, 49]. Supporting our findings, previous studies documented that the perceived level of and restrictions to participation affects the extent to which an individual feels included in their social circle, and the quantity of emotional resources the social circle can provide [50].

### Potential implications

Although this analysis has identified those of low SES to be vulnerable to loneliness, SES cannot generally be directly targeted by public health interventions [51]. Apart from broader interventions to address structural inequalities in health, functioning and participation, more focused interventions to address individual skills and resources are needed to avoid the gap between those less and more social disadvantaged. Mediation analysis can go further than purely descriptive analysis by identifying potentially modifiable targets of intervention on the pathway to loneliness, and help in understanding underlying mechanisms. The question that may now be posed is how this information can be used to tackle loneliness in a population of persons with a physical disability, with the end goal to improve health and wellbeing. As previously stated, the social gradient in health is not directly targeted by public health interventions, but rather addressed indirectly through the “health in all policies” directive [52], which may also contain initiatives to reduce participation restrictions for persons with physical disabilities and to strengthen psychosocial resources in this vulnerable population group. As we identified that low SES is followed by a chain of risk factors that contribute to the development of loneliness, it is highly relevant that persons with low SES are identified during the rehabilitation process, so that specific support and resources can be provided in order to reduce the potentially adverse effects of low SES on a wide range of mediators and ultimately loneliness. Routine assessment of SES during the rehabilitation process should take place without any ensuing stigma, but be understood as important social determinant of health. Health professionals may need to acknowledge that this vulnerable group with potentially lower health literacy needs intense support in the management of secondary health conditions and the maintenance of functional independence. Furthermore, specific interventions may be needed to support persons with low SES in overcoming restrictions to participation, and psychosocial interventions may help low SES groups in strengthening psychosocial resources, such as emotional support and self-efficacy. Potentially promising interventions for persons with physical disabilities that might ultimately reduce the negative effect of low SES on loneliness include interventions to enhance social support and social skills [53, 54], labour market participation [55], and participation more generally [56].

### Strengths and limitations

SwiSCI is a larger population-based study which provides a well-defined sampling frame and little sampling and response bias [23]. However, the cross-sectional nature of the data precludes inferences about causal relationships. This is especially problematic given that we attempt to compute mediation effects, why we restrict our conclusion to the discussion of interplay and associations between the multiple potential mediators, predictor and outcomes. We do however assume that the majority of socioeconomic variables would affect loneliness, and not vice versa. In order to address this issue of uncertain causality, future studies may use longitudinal data once it becomes available. The use of self-report data is also associated with recall and/or reporting bias as the reporting of health conditions, for example, cannot be validated by clinical data. Finally, variables may also be subject to reporting bias which can lead to spurious associations with loneliness. Loneliness is a broad concept which may overlap with several of the other variables understudy and may be reported, as with other psychological resources, as a shared effect of psychological personal factors. Furthermore, we restricted our analysis to variables suggested by the conceptual model and those available in our dataset. It is plausible that some of the associations exist due to unmeasured confounding or mediation. In light of these strengths and limitations, the main value of the evidence provided by the present study, is to identify vulnerable groups and highlight potential underlying mechanisms that necessitate further research.

## Conclusion

Our findings highlight the vulnerability of persons with low SES to loneliness in persons with SCI. Not only has this study emphasized the social gradient of loneliness, but it has also shown that an increased burden of secondary conditions and decreased functional independence, participation restrictions and the poor access to the psychosocial resources emotional support and self-efficacy lie on the pathway from low SES to loneliness. This population-based evidence suggests potential targets of interventions on the pathway to loneliness. The identified potential underlying mechanisms through which SES influences loneliness can be used to tailor comprehensive and interprofessional rehabilitative interventions focusing on the reduction of risk factors for loneliness in persons with SCI from lower SES groups.

## Data Availability

The datasets used and/or analysed during the current study are available from the corresponding author on reasonable request.

## Abbreviations

CI: Confidence interval
CFI: Comparative Fit Index
CHF: Swiss Francs
GSES: General Self-Efficacy Scale
IQR: Interquartile range
OECD: The Organisation for Economic Co-operation and Development
RMSEA: Root Mean Square Error of Approximation
SCI: Spinal cord injury
SCIM-SR: Spinal Cord Independence Measure for Self-Report
SCS-SCI: Secondary Conditions Scale for Spinal Cord Injury
SD: Standard deviation
SEM: Structural Equation Modelling
SES: Socioeconomic status
UCLA-SF: UCLA-short form
USER-P: Utrecht Scale of Evaluation of Rehabilitation-Participation
